# Long Standing Esophageal Perforation due to Foreign Body Impaction in Children: A Therapeutic Challenge in a Resource Limited Setting

**DOI:** 10.1155/2017/9208474

**Published:** 2017-08-08

**Authors:** Ngo Nonga Bernadette, Jean Jacques Ze, Angele O. Pondy, Claude M. Kalla, Nelly Kamgaing, Daniel Handy Eone

**Affiliations:** ^1^Department of Surgery and Anesthesiology, Faculty of Medicine and Biomedical Sciences, University of Yaoundé I, Yaoundé, Cameroon; ^2^Department of Medicine, Faculty of Medicine and Biomedical Sciences, University of Yaoundé I, Yaoundé, Cameroon; ^3^Department of Pediatrics, Faculty of Medicine and Biomedical Sciences, University of Yaoundé I, Yaoundé, Cameroon

## Abstract

Late presentation of foreign body impaction in the esophagus, complicated by perforation in children, has rarely been reported in the literature. Esophageal surgery is very difficult and challenging in Cameroon (a resource limited setting). We are reporting herein 2 cases of esophageal perforation in children seen very late (12 days and 40 days) after foreign body impaction, complicated with severe sepsis, who were successfully operated upon with very good results.

## 1. Introduction

Perforation of the esophagus is a well-recognized entity and can be spontaneous, traumatic, or iatrogenic. Foreign body ingestion is usually seen in children between 1 and 3 years of age and 10 to 20% will be impacted; most of them will be retrieved or removed without perforation [1]. Late presentation of esophageal perforation due to foreign body impaction in children has been rarely reported although foreign body is removed regularly from the esophagus of many children. The perforation may be life-threatening and would lead to severe mediastinitis, empyema, and sepsis with the expected high mortality. Sometimes the perforation may have minimal septic complications but leads to chronic tracheoesophageal fistula [2]. The management of thoracic esophageal perforation due to foreign body impaction has not been standardized because of insufficient data and experience. Very few cases of esophageal perforation complicated by severe sepsis due to foreign body impaction have been described in literature. The esophageal perforation has to be managed early, if the life of the patient is expected to be salvaged. No case of long standing severe sepsis due to esophageal perforation has been described in the literature. We are reporting 2 cases presenting with life-threatening conditions, following foreign body impaction, which were successfully managed in our service with the preservation of the native esophagus.

## 2. Case Report  1

**Figure 1:**
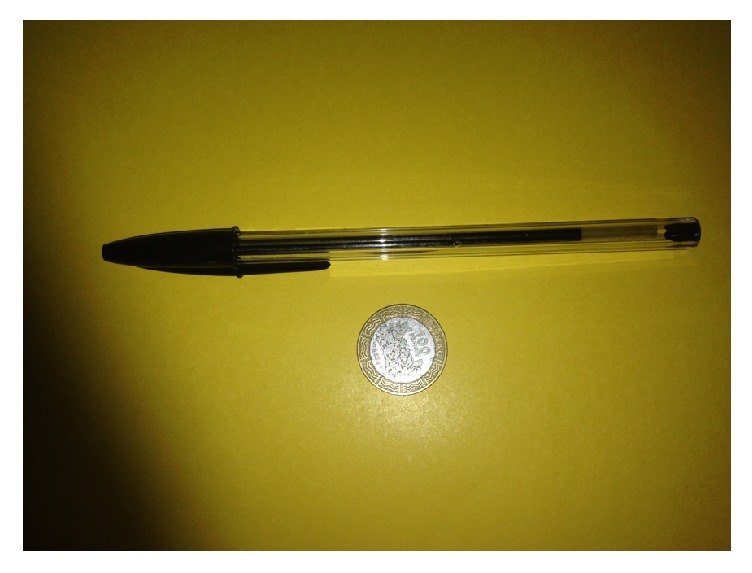
The type of impacted objects. A BIC pen and a 100 FCFA coin.

**Figure 2:**
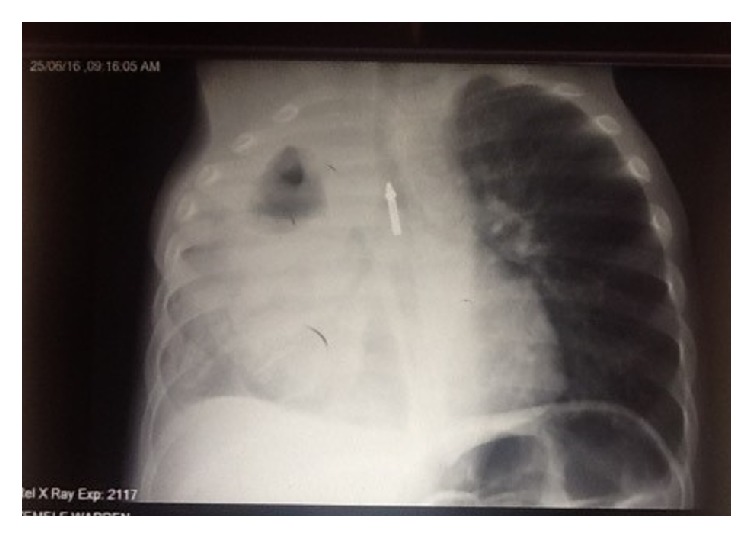
Chest X-ray of the boy at the time of the incident. The foreign body is visible in the chest. There is massive right pleural effusion and sign of esophageal perforation.

This was a 10-month-old boy, who was referred to us for empyema and sepsis, 40 days after esophageal perforation due to foreign body impaction. The mother reported that a little more than one month before admission, the child had swallowed a pen (BIC type 147 mm in length, Figure 1) and had been very sick ever since. Immediately the mother noticed drooling and respiratory distress, followed by fever and poor appetite; she carried him for consultation the next day at a health center, from where the child was referred to a major hospital. His chest X-ray revealed a foreign body in the esophagus and a massive right pleural effusion (Figure 2). The child was placed on antibiotics (ceftriaxone, amoxicillin/clavulanic acid, and metronidazole), and a chest tube was inserted. However, following the persistence of the state of ill health, he was referred 10 days later to two other major hospitals without improvement. There was a further deterioration of the child's condition, this time associated with feeding difficulties, weight loss on the persistent fever, and respiratory distress which were the reasons why he was brought to us (40 days following the incident).

**Figure 3:**
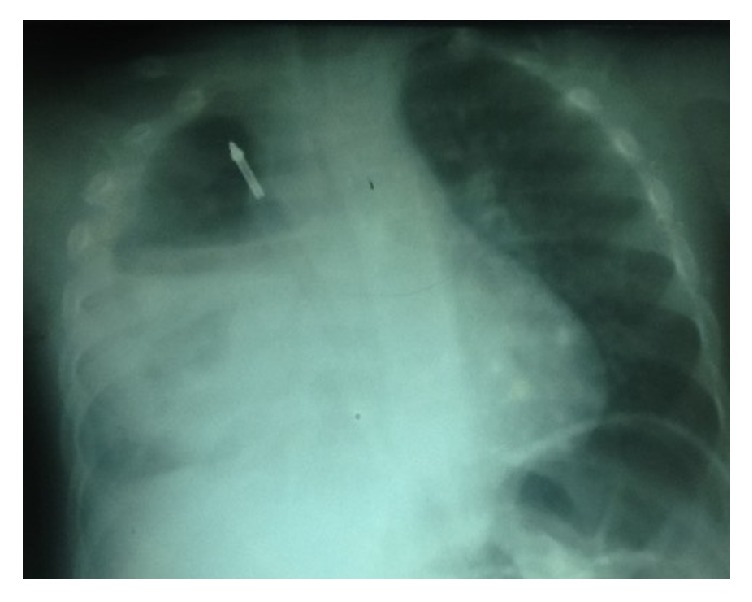
Chest X-ray one month later. This picture was taken the day of his arrival at our hospital. The empyema is loculated, the foreign body is still present in the chest, and the radiological signs of esophageal perforation are more obvious.

On arrival, the child looked ill, with respiratory distress and fever (39.5°C), and was in septic shock. The chest X-ray showed a massive loculated empyema on the right with an ipsilateral foreign body in the upper chest, which appeared to be in the esophagus (Figure 3). A complete blood count revealed a hyperleukocytosis of 42000/ml, moderate anemia with a hemoglobin level of 8.2 g/ml, and a normal platelet count. After a brief resuscitation, the child was taken to the operating room. A right posterior thoracotomy was conducted on the fifth intercostal space. We found a fibrinopurulent fluid and fibrinous empyema in the pleural space in association with false membranes and a 2 cm perforation on the anterior aspect of the esophagus just below the azygos vein, with the impacted foreign body in the lower esophagus. This foreign body (the pen) was oriented longitudinally and impacted in the esophagus and thus could not go through the lower esophageal sphincter. After a decortication, the foreign body was removed through the perforation. The esophageal tear was repaired by suturing, using a pleural flap, while a nasogastric tube was used as a stent. After thorough toileting, an indwelling drain was placed and this was then connected to our simplified drainage system as we have reported [3]. The surgery lasted 70 minutes in total. A nasogastric tube (NGT) was inserted for postoperative feeding. Postoperatively, he was placed on imipenem and metronidazole, considering he had received broad spectrum antibiotics for more than a month.

**Figure 4:**
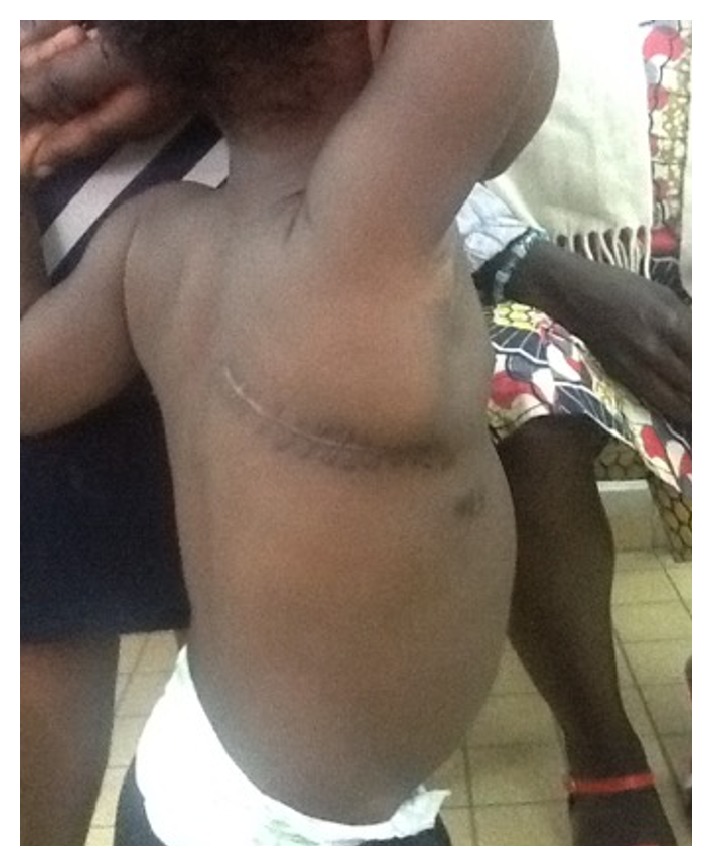
Surgical incision of the posterior thoracotomy in the baby boy one month after the surgery.

**Figure 5:**
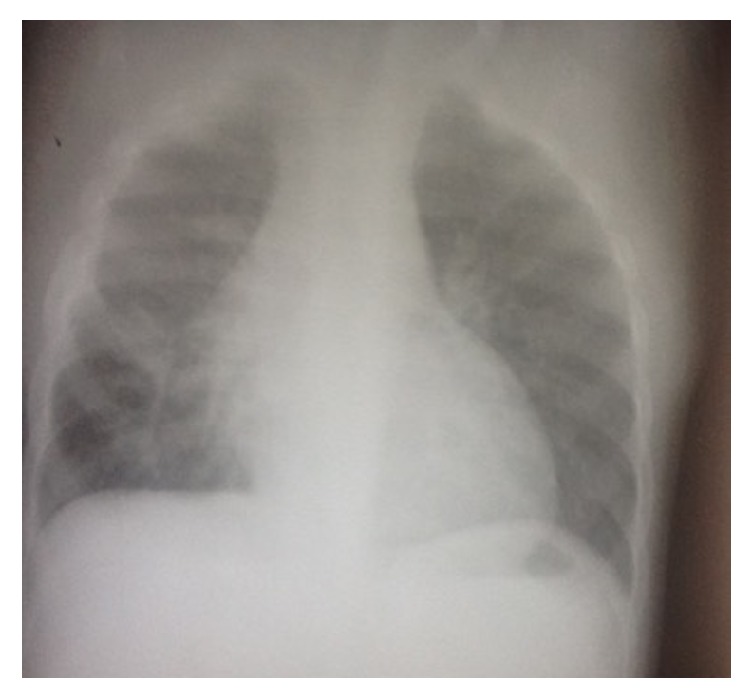
Chest X-ray, control one month after the surgery in the baby boy.

Feeding was begun on the day after surgery and this was exclusively via the NGT, for the first 6 days. Feeding by mouth was begun on the seventh day after surgery. The drain was removed on the fifth day and he was discharged 10 days after surgery. The postoperative course was uneventful. Figures 4 and 5 show, respectively, the posterior thoracotomy scar and the control chest X-ray at one month.

## 3. Case Report  2

**Figure 6:**
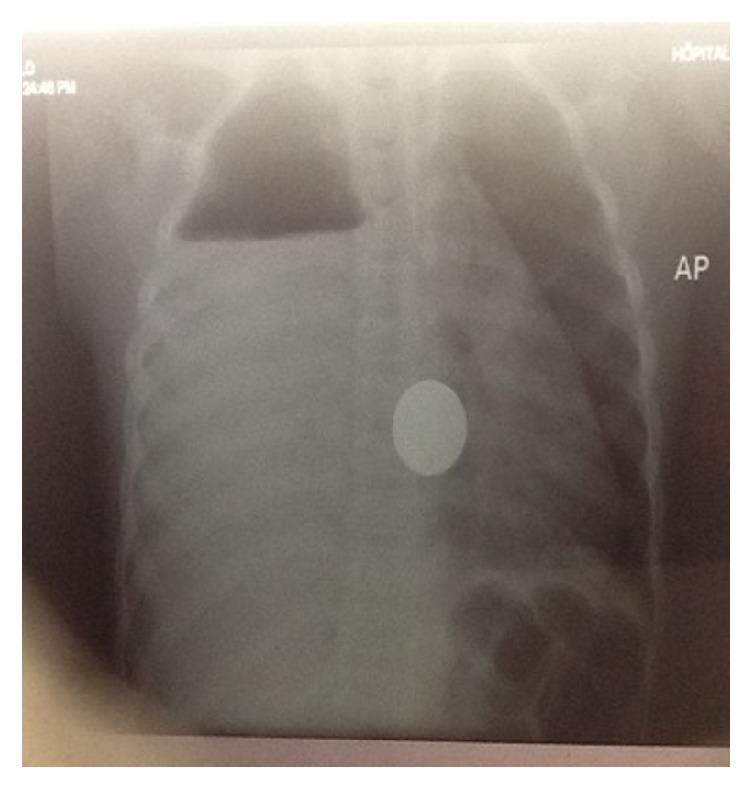
Chest X-ray of the 6 Y/O girl after the incident. Massive pleural effusion and air fluid level; foreign body in the right chest at the anatomical site of the esophagus and radiological signs of perforation.

**Figure 7:**
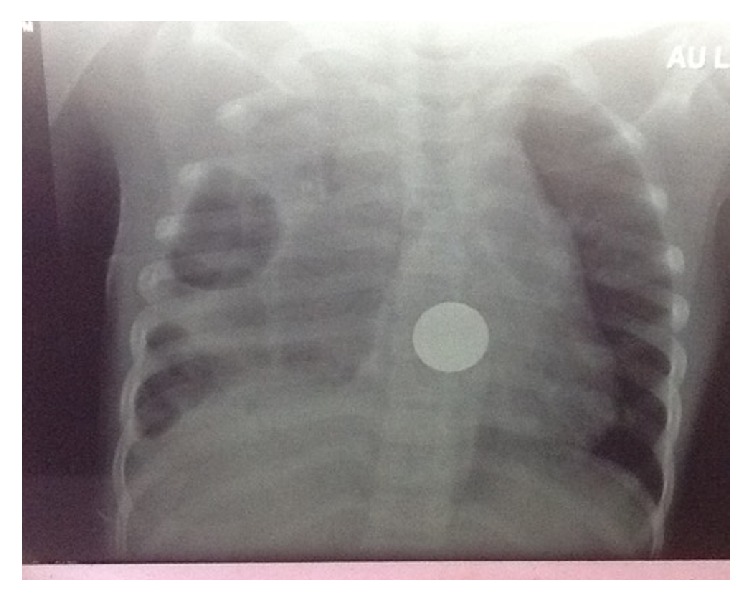
Chest X-ray of the same girl 12 days after admission, loculated empyema, and foreign body at almost the same position.

A 6-year-old girl, who happened to have swallowed a 100 FCFA coin (diameter 22 mm, Figure 1) on April 2014, was taken immediately to an ENT physician because of esophageal symptoms. An attempt (by the physician) to remove the coin with the aid of a rigid esophagoscope was unsuccessful and was rather complicated by perforation of the esophagus. The child was taken to a tertiary hospital in Douala (Cameroon) three days after the accident, where a chest radiograph and CT scan revealed a massive right empyema (Figure 6). A chest tube was placed and she was transferred to the intensive care unit, where she was placed on broad spectrum antibiotics. She was transferred to Yaoundé 12 days after the incident because her clinical state did not improve. On arrival, she was in severe respiratory distress and septic shock due to mediastinitis and empyema. The chest radiograph done on the day she arrived our casualty unit is shown in Figure 7. Immediate resuscitation was performed and the child was taken to the operating room, a few hours later. A right thoracotomy was done entering the chest at the level of the 5th intercostal space. Findings were massive empyema in the fibrinopurulent stage. The tear could not be located because of the magma of fibrin tissues. The coin was impacted just above the lower esophageal sphincter; thus it could not go through the sphincter. The esophagus was diffusely inflamed and as such very fragile. We removed as much fibrinopurulent tissue as we could and washed the pleural cavity until it became visibly clean. We then gently pushed the coin upwards and did a small longitudinal incision 3 cm below the azygos vein, through which the coin was removed. The hemodynamic condition of the child and our working environment did not permit for a longer procedure. We thus choose to be conservative. A nasogastric tube was inserted and then obstructed. A local pleural flap was used to buttress the sutured area. We washed the pleural cavity again and did a decortication. The corresponding lower lobe was rather inflammatory and void of pus. We left a single chest drain in place using the simple drainage system as we have reported [3]. The surgery took about 80 minutes from skin to skin. Postoperatively, she was taken back to the intensive care unit and placed on imipenem and metronidazole (the child had received amoxicillin/clavulanic acid, cefotaxime, and ceftriaxone in the preoperative period). Extubation was done on the same day of surgery and she was placed on 3 liters of nasal oxygen/min for 48 hours.

The patient was discharged from the intensive care unit on the 3rd day after surgery. The drainage remained purulent for 2 weeks. Daily care included antibiotics, high protein diets, respiratory physiotherapy, and wound care. She was fed exclusively through the NGT for the first 10 days after surgery. Oral feeding was resumed on the tenth day following surgery. However, the NGT tube was left in place for a further ten days for complementary feeding and removed on the twenty first day after surgery. She remained febrile for three weeks and was maintained on antibiotics. The drain was removed after 18 days. She stayed in the hospital for 1 month and was discharged on oral antibiotics after 5 days of restoration of strength and the absence of fever.

**Figure 8:**
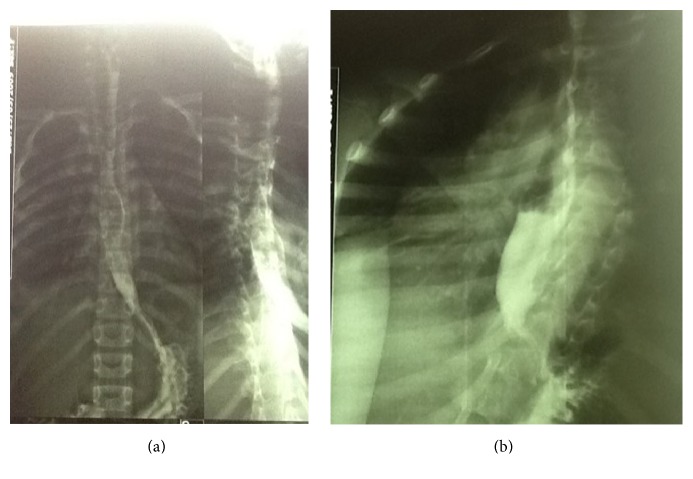
(a) and (b) Esophagogram of the girl 2 months after surgery. There are no leaks. The esophagus appeared normal.

**Figure 9:**
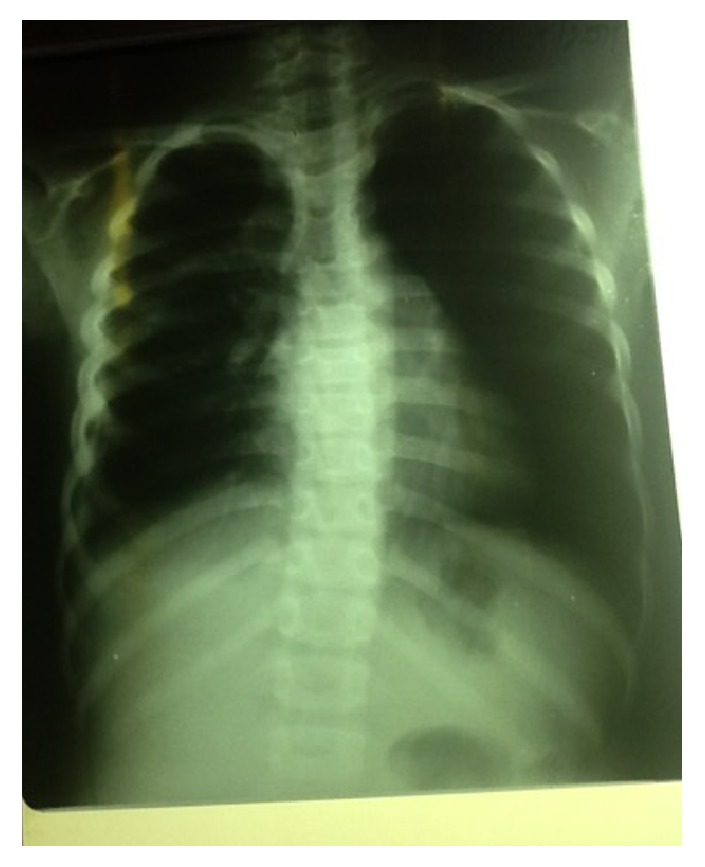
Chest X-ray: control at one year after both surgeries in the 6 Y/O girl.

She was followed up every week as an outpatient. Two weeks after discharge, she began running a low-grade intermittent fever for which we requested an esophagogram (Figures 8(a) and 8(b)). The findings were normal (and without any leak). She was readmitted 2 months after surgery because of the persistence of fever (this time of high-grade). A chest radiograph revealed a right lower lobe abscess. She was replaced on imipenem and was taken back to the operating room on the fifth day of admission because of the persistence of sepsis despite broad spectrum antibiotics. A thoracotomy, through the previous incision, was reconducted. We found a lower lobe abscess with destruction and gangrene of the right lower lobe. The esophagus was normal and without any signs of inflammation. We carried out just decortication and a lobectomy. The postoperative period was uneventful. Fever subsided on the day after surgery; meanwhile the drain was removed 5 days after surgery and she was discharged 10 days after the operation. She is followed up regularly as an outpatient. The chest radiograph one year after the surgery is shown on Figure 9 and is almost normal. She is currently healthy and doing well.

## 4. Discussion

Long standing esophageal perforation secondary to foreign body impaction in the chest and severe sepsis has not been reported in literature. Reported cases are those in children with localized tracheoesophageal fistula and inflammation after ingestion of battery [1, 2]. Impaction could result from just every object, with the commonest being coins and button battery. Sharp objects, although rare, could also be involved [1, 2, 4]. In western countries, children with esophageal perforation are readily managed at the time of diagnosis. In Cameroon, well-equipped hospitals and experienced surgeons for the esophagus are lacking. Surgeries of this nature are only done in our hospital, which also lacks appropriate infrastructures. Our practice is thus adjusted to match our environment.

Both children were in septic shock on arrival. The older girl was more sick, in severe hemodynamic compromise, and about to give up. We saved her life after extensive reanimation. This accounted for her longer stay in the hospital and possibly why she was operated upon twice. The lower lobe abscess was a delayed complication of the severe sepsis and the extensive inflammation we noticed in the right chest during the first surgery. There was no indication to do a lobectomy at that time. The right chest was the site of an extensive inflammation and infection involving all the surfaces: esophagus, pleura, and visceral pleura. All pulmonary lobes were deeply inflamed and embedded in a fibrinopurulent tissue resulting in stiffness.

The type of foreign body may be important, although both objects were obstructive. The coin in the six-year-old girl (diameter 22 mm) was more obstructive and might have been the reason for the more severe infection of the pleural cavity. The small boy was still able to swallow some liquids although with difficulties. We believe this is the reason why the perforation was evident even 40 days later.

It is very unusual for a child with esophageal perforation complicated by mediastinitis, empyema, and sepsis to survive for more than ten days without surgery. The presence of the impacted foreign body in the thoracic esophagus that continued to leak in the chest resulted in the continuous sepsis. No guidelines exist for cases of this nature. It is difficult to predict what would be the best treatment. Recently conservative management has become more common in children after perforation following dilatation [1, 2]. Long standing severe sepsis due to esophageal perforation following foreign body impaction is a life-threatening condition, in which the esophagus, as well as surrounding tissues, becomes heavily inflamed, thus rendering any dissection hazardous and dangerous. This was the case in both children and we felt that any attempt to dissect the esophagus could have been very risky. More so, the hemodynamic condition of the children would not have permitted for a longer procedure (none of the procedures lasted more than 90 minutes.). We chose then to wash, repair, and stent the diseased esophagus after removing the foreign body. This decision was successful as both children survived. Other factors which contributed to the success were early feeding and the use of imipenem (directed against the sepsis). We believe that children's esophagus is more elastic and sometimes they just need a little help to recover.

The management of esophageal perforation in children has become more conservative and depends on the cause of perforation, its location, the integrity of the esophagus, and time elapsed [2, 5].

The results of the treatment applied have been variable.

Ramareddy and Alladi reported 4 cases of perforation secondary to foreign body impaction for which they did medical treatment in 2, diversion in 1, and drainage in 1 patient. To note is that these cases presented early and are thus not as our patients. Two of their cases presented with perforation at the cervical esophagus and none was thoracic [1].

Sarin and Sinha observed 11 cases of perforation in children with a mortality of 36% and a prolonged hospital stay (80 days) and recommended a more aggressive approach [6].

Al Jubab et al. after reviewing esophageal perforation reported that the best prognosis for perforation was observed for cases seen at an early stage and recommended that late cases of perforation should be observed provided there is no empyema or mediastinitis [7]. We agree with them that observation is not an option in the phase of severe sepsis due to extensive empyema or mediastinitis. The question is what is to be done with an inflamed esophagus in a septic child? We think that, in cases of extreme sepsis and longstanding perforation with ongoing severe sepsis, the best option is to remove the foreign body, wash the pleural cavity, and repair and splint the esophagus. Children's esophagus is often more elastic and thus recovers quicker than adults.

Peters et al. reported 7 cases of perforation following impaction in children of which 5 of those perforations were thoracic. These cases were managed surgically without esophagectomy and recorded no deaths. Their series did not present with empyema or mediastinitis. That is the only study reporting similar cases to ours [8]

Altokhais et al. did a review of foreign body impaction from 1995 to 2013 and found 4 complications in 70 patients, some of which were chronic and did not have hemodynamic compromise like our patients. Most of the patients presented with coins as foreign body. There were cases of fistula secondary to battery ingestion. The case that harbored inflammation the most was operated on 25 days after the ingestion of the foreign body. This inflammation, however, was well localized and without massive empyema or septic shock. They carried out a thoracotomy, conservative management, and stenting without any complications [2].

## 5. Conclusion

For long standing esophageal perforation in the chest, we will recommend saving the native esophagus (if there is no gangrene of the esophagus) repairing the perforation, splinting the esophagus, enteral nutrition as soon as possible through the nasogastric tube, and broad spectrum antibiotics. Children's esophagus is elastic and heals quicker than adults. More experience is needed to define strict guidelines as experience is lacking for such cases owing to their rarity.
